# Is the Quality of Toothache-Related Information Published in Brazilian Websites Adequate to Assist People in Seeking Dental Treatment?

**DOI:** 10.3290/j.ohpd.a44142

**Published:** 2020-07-04

**Authors:** Matheus Lotto, Patricia Estefania Ayala Aguirre, Natalino Lourenço Neto, Agnes Fátima Cruvinel, Thiago Cruvinel

**Affiliations:** ^a^ Dentist, Department of Pediatric Dentistry, Orthodontics and Public Health, Bauru School of Dentistry, University of São Paulo Al. Dr. Octávio Pinheiro Brisolla, 9–75, Vila Universitária, 17012-901, Bauru, SP, Brazil. Contributed to conception and design, data acquisition, drafted and critically revised the manuscript.; ^b^ Dentist, Department of Pediatric Dentistry, Orthodontics and Public Health, Bauru School of Dentistry, University of São Paulo Al. Dr. Octávio Pinheiro Brisolla, 9–75, Vila Universitária, 17012-901, Bauru, SP, Brazil. Contributed to conception and design, data acquisition, drafted and critically revised the manuscript.; ^c^ Professor, Department of Pediatric Dentistry, Orthodontics and Public Health, Bauru School of Dentistry, University of São Paulo Al. Dr. Octávio Pinheiro Brisolla, 9–75, Vila Universitária, 17012-901, Bauru, SP, Brazil. Contributed to data interpretation and critically revised the manuscript.; ^d^ Professor, Discipline of Public Health, School of Medicine, Federal University of Fronteira Sul, Rodovia SC 484 – Km 02, Fronteira Sul, 89815-899, Chapecó, SC, Brazil. Contributed to data interpretation and critically revised the manuscript.; ^e^ Professor, Department of Pediatric Dentistry, Orthodontics and Public Health, Bauru School of Dentistry, University of São Paulo Al. Dr. Octávio Pinheiro Brisolla, 9–75, Vila Universitária, 17012-901, Bauru, SP, Brazil. Contributed to conception and design, data analysis and interpretation, drafted and critically revised the manuscript.

**Keywords:** toothache, eHealth, medical informatics, consumer health information

## Abstract

**Purpose:**

The aim of this study was to evaluate the readability and the quality of toothache-related information found in Brazilian websites.

**Materials and Methods:**

Fifty-five websites retrieved from Google Search, Baidu, Yahoo! and Bing were evaluated by two independent examiners using the DISCERN questionnaire, the *Journal of American Medical Association* *(JAMA)* benchmark criteria and the Flesch Reading Ease adapted to Brazilian Portuguese (FRE-BP). Additionally, the websites were categorised according to their information, adopting four criteria related to: (i) endodontic pain, (ii) toothache relief or treatment, (iii) the self-resolution of pain, and (iv) the promotion of home remedies usage. The statistical analysis was performed using Spearman’s rank correlation coefficient, Mann–Whitney U test, hierarchical clustering analysis by Ward’s minimum variance method, Kruskal–Wallis test, post-hoc Dunn’s test and Chi-square test. P < 0.05 was considered statistically significant.

**Results:**

The overall means (± SD) of DISCERN and FRE-BP were, respectively, 31.02 (± 5.56) and 61.20 (± 11.79), without quality-based differences between the websites with health- and non-health-related authors, and distinct clusters.

**Conclusion:**

Therefore, the quality of toothache-related information found in this sample of Brazilian websites was classified as simple, accessible and of poor quality, which can hamper the personal decision-making process of seeking dental treatment, leading to damages caused by the non-effective self-management of toothache.

The dominance of biomedical model of health, focused on curative procedures guided uniquely by the application of the biological knowledge, is decreasing over time towards a participative healthcare. This paradigm shift is supported by three main causes: the ageing of population, the increase in non-communicable diseases, and higher levels of clinical costs.^39^ In this context, people drive their efforts to the management of their own conditions^23^ by the adoption of proper actions about their lives, such as the consumption of health information. This subjective phenomenon is originated from the primary concept of ‘the care of the self’ as an impulse of the intrinsic need of human existence.^25^

Simultaneously, information and communication technologies (ICTs) are evolving and disseminating worldwide, with a special attention to the internet.^2,27,36^ This media is increasingly being employed as a complementary source of health information,^44^ ranking this topic as the second interest among Google users.^53^ The utility in understanding these digital behaviours for supporting the actions of public health planners, therefore, stimulated the raising of two novel research fields – infodemiology and infoveillance – both coined by Gunther Eysenbach. While infodemiology is defined as ‘the science of distribution and determinants of information in an electronic medium, specifically the internet, with the ultimate aim to inform public health and public policy’,^20,21^ infoveillance is characterised by the use of infodemiology data for health surveillance purposes.^20,21^ These disciplines proved to be useful for the better comprehension of people’s behaviours in relation to infectious diseases, epilepsy, cancer, multiple sclerosis and so forth.^9,10,36,62^

Taking into account the opinion of patients, health-seeking behaviours aid in the patient–health professional relationship through the improvement of layperson’s skills,^60^ such as the capacity of forming questions, effective communication and a better understanding of professional counseling.^12^ Although the progress of this complex scenario favours preventive attitudes, a considerable number of individuals are still unaware of their health conditions, only reacting in the presence of disease symptoms.^28^ In this sense, the onset of toothache plays an important role on the emergence of a late consciousness of oral problems, especially when considering dental caries.^13^ Since toothache markedly affects the activities of daily living, work performance and productivity, and quality of life,^35^ it compels people to search for strategies related to relief, treatment or self-resolution of pain.^15,36^

Indeed, the internet presents a great potential to improve health education and experiences of population^5,46^; however, recent studies have shown that health information usually found online is of poor quality^3,6,34^ and/or high reading difficulty levels.^1^ In these situations, websites may hamper or delay people’s decision-making processes in seeking treatment; eg, 35% of Americans who searched health issues on the internet admitted they did not seek professional care due to digital recommendations.^46^ Additionally, it is noteworthy that factors influencing online consumers in the establishment of perceived quality and adequacy of information are based on empirical characteristics, such as the experience of other users, design, complexity and style of information.^3^ This pattern is still more common among individuals with low health literacy levels.^4,8^

Considering three findings related to Brazil in comparison with developed countries, (i) higher prevalence of toothache mainly among vulnerable groups,^48^ (ii) lower education levels, with 27% of functional illiterates^14,45^ and 71% of individuals with low health literacy,^8^ and (iii) an increased penetration of the internet over the last years, exceeding more than 65% of population,^56^ the evidence about parameters of toothache-related written content published in Brazilian websites is *sine qua non* for the better understanding of their impact on the maintenance and improvement of oral health status of internet users,^63^ contributing to the development of policies for the production of educative materials, and guidelines for the instruction of patients. Novel approaches to the reduction of consequences of dental pain are quite desirable global goals for oral health in 2020.^30^

Therefore, the aim of this study was to evaluate the readability and the quality of toothache-related information found on Brazilian websites.

## MATERIALS AND METHODS

### Study Design

This study used the methodology proposed by Aguirre et al (2017).^3^ The quality of information about the toothache was analysed in Brazilian websites. After the development of a specific search strategy, the websites were retrieved from the following search engines: Google Search, Baidu, Yahoo! and Bing. Duplicates, non-specific, inaccessible and/or scientific links were excluded. The websites were evaluated by two independent examiners using the DISCERN questionnaire,^17^ the *Journal of American Medical Association (JAMA)* benchmark criteria,^49^ and the Flesch Reading Ease adapted to Brazilian Portuguese (FRE-BP).^37^

### Search Strategy

The search strategy was designed from the most relevant terms used on the internet. Initially, the term *toothache* in Brazilian Portuguese was entered in Keyword Planner, in order to list the automatic matches available. Then, the relevance of each one of these terms was analysed on Google Trends, observing the variation of their Relative Search Volume (RSV) in the period from 2004 through 2015, including all categories of internet queries performed in Brazil. After the exclusion of terms that did not have statistically significant search volume, a final search strategy was developed by the combination of three terms (‘dor de dente’ + ‘dor dente’ + ‘dente doi’), which correspond to the synonyms and typos of toothache written in Brazilian Portuguese.

### Selection of Websites

The websites were selected through the four search engines with the largest market share worldwide: Google Search, Baidu, Yahoo! and Bing.^42^ The searches were performed in a computer connected to the internet, using browsers with deleted histories and cookies, set up to retrieve only websites published in Brazilian Portuguese and accessed in Brazil.

Subsequently, the websites were registered using WebCite (WebCite Consortium, Toronto, Canada),^61^ an online service that archives the information exactly as it was recovered, avoiding changes and updates for future analyses.

After that, the websites were dichotomised according to the nature of their authorship, by health-related or non-health-related authors. The websites or blogs developed by dental or medical associations, universities, educational institutions, health companies or health professionals were classified as health-related authors, while all of other cases were classified as non-health-related authors. Additionally, the information presented on the websites was categorised according to four criteria: (i) related to endodontic pain (no/yes); (ii) related to relief or treatment of toothache (no/yes); (iii) related to self-resolution of pain (no/yes); and (iv) related to the home remedies usage for controlling toothache (no/yes). The presence or absence of these contents was graphically represented by the software Genesis version 1.7.7 (Institute of Computational Biotechnology, Graz, Austria), characterising the identity of each website.^41,52^

### The Assessment of Quality of Websites

Two independent examiners evaluated the quality of websites using the DISCERN questionnaire^17^ and *JAMA* benchmark criteria.^49^ The DISCERN questionnaire is usually applied to access the quality of written information on health treatment choices. The instrument is divided into the following three sections: (1) reliability of publication; (2) specific details of the information about treatment choices; and (3) overall quality rating of the document. It consists of 16 questions with 5-level Likert scale, where the score ‘1’ indicates that the criterion was not accomplished and the score ‘5’ indicates that the criterion was completely satisfied. The total DISCERN score varies between 15 and 80, since the second question must be disregarded when the first question is scored ‘1’. Typically, only the results of the first and second sections of the instrument were used to qualify the health content of documents, as follows: very poor (15–26), poor (27–38), fair (39–50), good (51–62), and excellent (63–75).^29^ The third section was used to confirm the coherence of investigators over the analysis.

The *JAMA* benchmark consists of a series of four qualitative criteria that refer to the description of the authorship (author’s name, affiliations, and credentials), attribution (effective references of content), currency (presence of dates of posts and update of information), and disclosure (the statement of any potential conflicts of interests) of websites. One point is given for each fulfilled criterion, with a total score varying from 0 to 4.

The websites that were divergently qualified by the examiners were re-assessed until the achievement of a consensus score.

### Readability Measures

The FRE-BP^37^ was used to assess the readability of the websites based on the following formula:

FRE-BP = 248.835−(84.6 × syllables per word) − (1.015 × words per sentence).

These metrics were calculated using the online tool Readable.io (Readable.io, Bolney, England),^47^ considering the information related to the respective Uniform Resource Locator (URL) of each website. All analyses were performed based on the overall written content downloaded from these links. The reading difficulty of a written material is presented according to the following scores: very easy (75–100), easy (50–75), difficult (25–50), and very difficult (0–25).

### Statistical Analysis

Data were analysed with the Statistical Package for Social Science version 21.0 (SPSS, Chicago, USA). After the rejection of the hypothesis of normal distribution of data by Kolmogorov–Smirnov test, the statistical analysis was performed by the application of non-parametric tests. The internal consistencies of DISCERN and *JAMA* benchmark were determined by Cronbach’s alpha. The correlations between distinct measures were demonstrated by Spearman rank correlation coefficients. The statistically significant differences between the dichotomised natures of websites were observed by Mann-Whitney U test. The clusters that emerged from the similarity of websites identities were determined by the hierarchical clustering analysis using the Ward minimum variance method. Distinct clusters were compared by Kruskal–Wallis and post-hoc Dunn’s test. The differences in the distribution of clusters in relation to the quality (DISCERN < 39 vs DISCERN ≥ 39) and natures (health- vs non-health-related authors) of websites were evaluated by Chi-square test. P values < 0.05 were considered statistically significant for all analyses.

## RESULTS

### Websites

**Fig 1:**
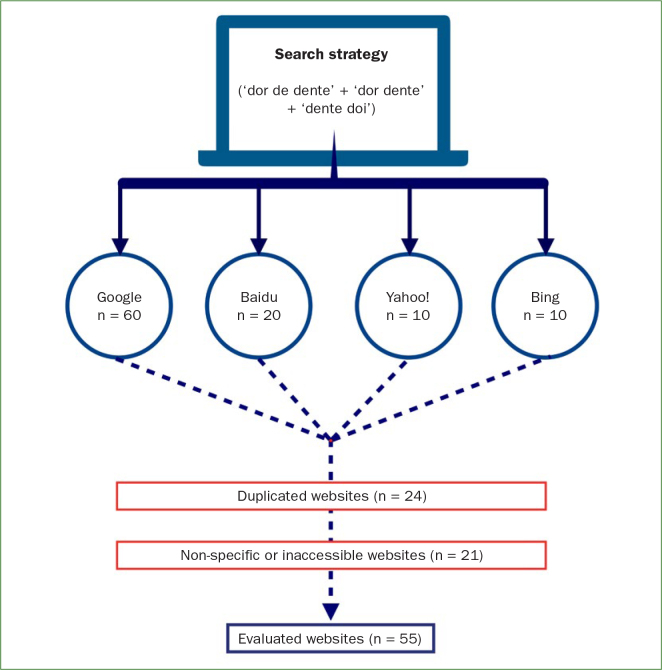
Flowchart depicting the systematic selection of Brazilian websites containing tooth pain information.

A hundred websites were obtained from the first links retrieved in sequence by Google Search (n = 60), Baidu (n = 20), Yahoo! (n = 10) and Bing (n = 10). Duplicate websites (n = 24) and non-specific or inaccessible ones (n = 21) were excluded. Fifty-five websites met the inclusion criteria for this analysis, as shown in Figure 1.

### Reliability of Instruments

The DISCERN features excellent internal consistency (Cronbach’s alpha = 0.804, 95% CI:0.719–0.872), whereas the *JAMA* benchmark criteria showed a low internal consistency (Cronbach’s alpha = 0.345, 95% CI: 0.009–0.588).

### DISCERN, JAMA, and FRE-BP Scores

Toothache-related websites available for Brazilian population presented poor quality of information considering the mean and median of DISCERN scores (Table 1). The scores obtained by the sum of the first 15 questions of DISCERN (S1 + S2) were significantly correlated with the score of the question 16 (p = 0.624, p < 0.001), differently of that observed between DISCERN and *JAMA* benchmark (p = 0.015, p = 0.270).

**Table 1:** Descriptive statistics of DISCERN, *JAMA* benchmark and FRE-BP scores

	S1	S2	S3	DISCERN (S1 + S2)	JAMA	FRE-BP
Mean	17.78	13.24	2.51	31.02	1.67	61.20
SD	2.75	4.47	0.72	5.56	1.10	11.79
Median	18.00	14.00	2.00	32.00	2.00	62.17
Minimum	12.00	7.00	1.00	20.00	0.00	21.88
Maximum	26.00	22.00	4.00	43.00	4.00	85.59

S1, S2, and S3 = three different sections of DISCERN. FRE-BP = Flesch Reading Ease adapted to Brazilian Portuguese.

The highest quality score (DISCERN = 43) was found in a dental blog specialised in answering recurrent doubts of dental health seekers (www.medodedentista.com.br/). A total of 12 websites (21.8%) presented fair quality (DISCERN ≥ 39), while other 12 websites scored *JAMA* benchmark ≥ 3, with most of them satisfying only one criterion (n = 35). While most pages described their authorship (60%) and disclosure (50.9%), the minor percentage of them made available their attribution (23.6%) and currency (32.7%).

In accordance to FRE-BP scores, websites were considered simple and accessible for most population (Table 1), although the reading grade level of websites was not correlated with DISCERN (p = –0.009, p = 0.949) and *JAMA* benchmark (p = –0.002, p = 0.99).

The quality of websites developed by health- or non-health-related authors was considered statistically similar considering both instruments, DISCERN (p = 0.58) and *JAMA* benchmark (p = 0.53) (Table 2). Also, the reading grade levels found in websites produced by health- and non-health-related authors were similar (p = 0.46).

**Table 2:** Descriptive statistics of websites with health- and non-health-related authors for DISCERN, *JAMA* benchmark and FRE-BP. Different lowercase letters mean statistically significant differences between groups (Mann–Whitney U test, p < 0.05)

Websites		S1	S2	S3	DISCERN (S1 + S2)	JAMA	FRE-BP
Health-related authors (n = 34)	Mean	17.76^a^	13.59^a^	2.44^a^	31.35^a^	1.56^a^	62.12^a^
	SD	2.49	4.60	0.66	5.67	0.99	12.60
	Median	18.00	14.00	2.00	32.00	1.50	62.68
	Minimum	14.00	7.00	1.00	22.00	0.00	21.87
	Maximum	22.00	22.00	4.00	43.00	3.00	85.59
Non-health-related authors (n = 21)	Mean	17.81^a^	12.67^a^	2.82^a^	30.48^a^	1.86^a^	59.68^a^
	SD	3.19	4.69	0.80	5.48	1.27	10.42
	Median	17.00	13.00	3.00	31.00	2.00	60.35
	Minimum	12.00	7.00	1.00	20.00	0.00	31.31
	Maximum	26.00	19.00	4.00	40.00	4.00	74.08

S1, S2, and S3 = three different sections of DISCERN. FRE-BP = Flesch Reading Ease adapted to Brazilian Portuguese.

### Website ID

**Fig 2:**
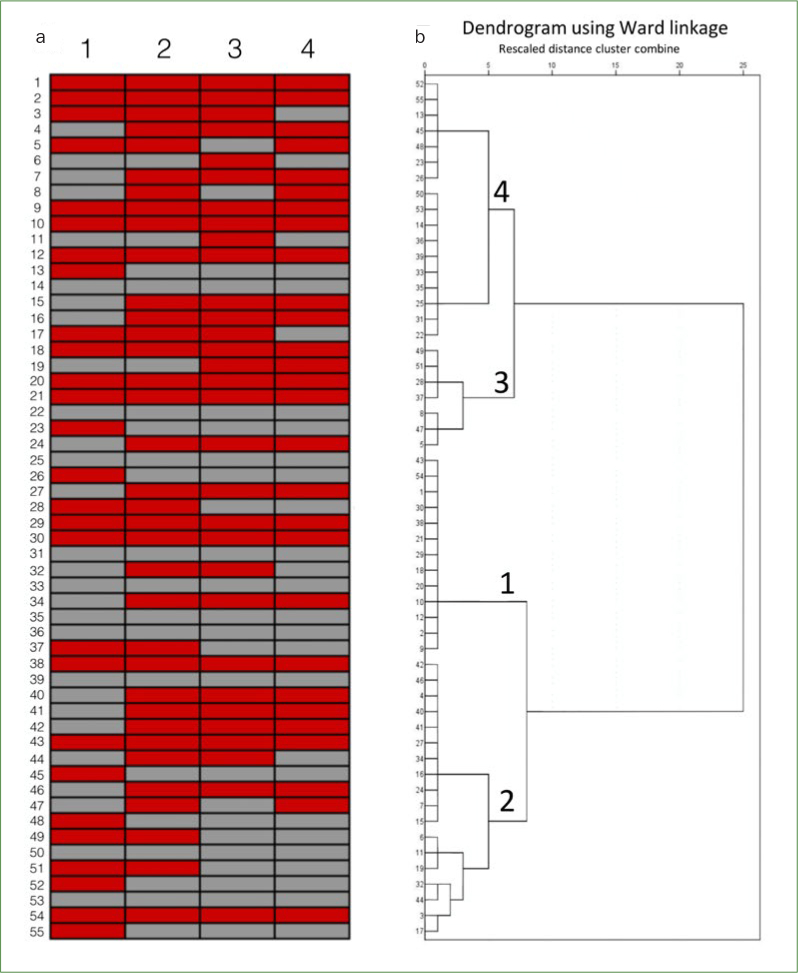
Cluster analysis of the websites. (a) The representation of websites’ IDs regarding the content of information: related to endodontic pain (1), related to relief or treatment of toothache (2), related to self-resolution of pain (3), and related to the home remedies usage for controlling toothache (4). Red and grey bars mean the presence and absence of the information, respectively. (b) Dendrogram depicts four clusters originated from the websites’ IDs (hierarchical clustering analysis by Ward’s minimum variance method).

As shown in Figure 2, the hierarchical clustering analysis generated four categories of websites based on the presence or absence of specific contents. There were no differences between clusters in relation to the quality and natures of websites (Chi-square, p > 0.05). The percentages of websites with health-related authors were 69.2% for cluster 1, 44.4% for cluster 2, 71.4% for cluster 3, and 70.6% for cluster 4. Websites that mentioned ≤ 1 of specific contents (cluster 4) presented a higher *JAMA* benchmark average score in comparison with websites that mentioned two (cluster 3, p = 0.02) and four (cluster 1, p = 0.01) specific contents (Table 3).

**Table 3:** Descriptive statistics of different clusters of websites for DISCERN, *JAMA* benchmark and FRE-BP. Different lower case letters mean statistically significant differences between the groups (Kruskal–Wallis test and post-hoc Dunn’s test, p < 0.05)

Cluster		This media is increasingly being employed S1	S2	S3	DISCERN (S1 + S2)	JAMA	FRE-BP
**1** **(n = 13)**	Mean	17.31^a^	13.38^a^	2.31^a^	30.69^a^	1.23^a^	65.49^a^
	SD	2.13	4.94	0.48	6.56	1.23	9.50
	Median	17.00	14.00	2.00	34.00	1.00	66.24
	Lower	16.02	10.40	2.02	26.73	0.48	59.75
	Upper	18.60	16.37	2.60	34.66	1.98	71.24
	Interquartile	4.00	9.00	1.00	12.00	2.00	11.78
**2** **(n = 18)**	Mean	17.44^a^	12.72^a^	2.39^a^	30.17^a^	1.56^a^,^b^	59.97^a^
	SD	2.14	3.78	0.85	5.50	1.04	9.95
	Median	17.00	13.50	2.00	34.00	1.00	61.30
	Lower	16.38	10.84	1.97	27.43	1.04	55.02
	Upper	18.51	14.60	2.81	32.90	2.07	64.90
	Interquartile	3.00	5.00	1.00	9.00	1.00	9.73
**3** **(n = 7)**	Mean	18.00^a^	16.71^a^	2.71^a^	34.71^a^	1.00^a^	56.48^a^
	SD	2.70	2.13	0.75	2.56	0.57	8.66
	Median	18.00	17.00	3.00	35.00	1.00	57.57
	Lower	15.50	14.74	2.02	32.34	0.47	48.48
	Upper	20.50	18.69	3.41	37.09	1.53	64.50
	Interquartile	6.00	3.00	1.00	3.00	0.00	10.08
**4** **(n = 17)**	Mean	18.41^a^	12.24^a^	2.71^a^	30.65^a^	2.41^b^	61.14^a^
	SD	3.69	5.04	0.68	5.52	0.87	15.52
	Median	19.00	10.00	3.00	30.00	2.00	62.17
	Lower	16.51	9.64	2.35	27.81	1.96	53.16
	Upper	20.31	14.83	3.06	33.49	2.86	61.13
	Interquartile	7.00	9.00	1.00	9.00	1.00	15.83

S1, S2, and S3 = three different sections of DISCERN. FRE-BP = Flesch Reading Ease adapted to Brazilian Portuguese.

## DISCUSSION

To our knowledge this is the first study that evaluated the quality of toothache-related information available on Brazilian websites. These findings showed a pattern of low-quality information, with only 21.8% of websites presenting acceptable contents, in accordance with the DISCERN instrument (≥ 39). Also, the websites were considered easy and accessible for most population when regarding FRE-BP scores. These results are in agreement with studies that also evaluated the quality of health information in digital sources.^6,16,34^ In this context, the influence of ICTs on dental education is a cause of concern for the maintenance of oral health status in different populations, especially among people in vulnerable social conditions, more affected by untreated oral diseases; hence, they are more susceptible in seeking for self-resolution of dental pain, such as home remedies and other miraculous measures.^38^

As shown in Figure 2, most websites published information about self-resolution (n = 31) and home remedies usage for controlling toothache (n = 28), reflecting the need of internet users for obtaining orientation to relieve their own symptoms. This fact may be related to multiple circumstances, such as the high cost dental procedures,^38^ the low per capita income found in Brazil,^11^ the inefficiency of public health system,^32^ the desire of people in solving their oral health problems guided by ICTs,^33^ and the low level of health literacy of Brazilian population.^8^

The easy access to information predisposes people to seek advices from the internet,^18^ mainly individuals of new generations.^58^ In Brazil, the internet penetration increased from 2.87% in 2000 to 60.87% in 2016,^55^ with projections of 2% of growth until 2022.^51^ This number represents nearly 125 million of users, being the largest internet market in Latin America and the fourth most in the world.^57^ Therefore, dentists should take advantage of ICTs for counselling their patients. Differently of laypersons, however, health specialists consider that internet hampered the relationship with their patients, concerning about the quality, relevance and consequences of online information. In this sense, three main attitudes are instinctively taken by professionals when they are confronted with internet-based health issues: (a) they react defensively demonstrating their expertise, or (b) they instruct patients in analysing contents adequately, or (c) they guide people in looking for reliable sources of information.^54^ Although the latter two reactions are positive to the shared decision-making process, these conditions might not be sufficient for increasing the criticism of individuals. Few patients are proactive in discussing their interests with health professionals. Then, dentists should train their patients in accessing specific websites to obtain validated information, besides teaching mechanisms for the selection of adequate contents, such as triangulation.^40^ This technique consists in a cross-checking procedure for assessing the regularity of data among multiple sources of information,^43^ providing a more detailed and balanced picture of a given situation.^7^ The lack of professional assistance in this process could generate conflict and patients’ distrust, leading them to the acceptance of online advice and refusal of expert counseling.^50^

The concomitant application of two instruments for measuring the quality of information was important to increase the discrimination of websites, since the results yielded by both tools were not correlated. The structure of *JAMA* benchmark analyses editorial aspects that denote the responsibility of authors in producing reliable materials, while DISCERN is focused on the critical appraisal of written health information. Only two websites presented satisfactory DISCERN and JAMA scores simultaneously, of which one website published information about the management of pain related to the eruption of third molars, and the other instructed people on first aid in toothache. In the latter case, authors highlighted a statistically significant caveat: ‘and the most important – painkillers, compresses or anything else that your neighbour had suggested to you will be effective, at maximum, as a palliative measure. If your tooth hurts, run to the dentist. This is the solution.’ This sentence denotes the authors’ consciousness on the influence of Web contents on health seekers. Surprisingly, no statistically significant differences were observed between health- and non-health-related authors in relation to DISCERN and JAMA scores (Table 2), which indicates a lack of commitment of health professionals in producing good quality educational materials for digital publication. Previous studies have already shown that health professionals were frequently not engaged with the production of good quality and accessible information.^22,54^

The legibility of websites indicates the availability of simple and accessible information on toothache in Brazilian websites. The non-correlation between FRE-BP and DISCERN demonstrated that (i) the assessment of quality of information was not influenced by the reading difficulty levels of websites, and (ii) the adequacy of health contents was not affected by their eruditeness or by the construction of intricate texts. As the websites were evaluated only by professionals with higher education levels, the writing complexity of materials might contribute to a possible bias of false-perception of quality, which was discarded by the present results, corroborating an evidence that showed no statistically significant differences between DISCERN scores obtained by laypersons and specialists.^59^ It is crucial to note that dental knowledge raised from the consumption of digital information depends on the dialectical interaction between a person and a computer, ie, low levels of health literacy difficult the understanding and interpretation of oral health-related publications, impacting negatively on the self-perception of dental treatment needs, and the frequency of dental visits.^31^

The internet promoted the electronic dissolution of geographical borders and, consequently, the effervescence of heterogeneous interactions of people living in different social contexts, such as freedom and repression, war and peace, democracy and tyranny, etc. This virtual environment is characterised by an open and asynchronous media, which favours the identity expression by the privacy preservation. Since it is almost ubiquitous, the internet strongly influences people’s behaviours, thoughts, feelings and actions. The availability of information combined with the intellectual activity of ‘producing and expressing own opinion’ contributes to the construction of health values of individuals based on ‘the care of the self’.^25^ In view of this, people require their active participation in healthcare, even in a latent form. The shared decision-making process becomes an essential value for the adherence and engagement of people in preventive and treatment strategies. Hence, disregard these facts that transcend the practice, the formation and the opinion of professionals might put the dentist under the risk of working without intervening, of practising without any purpose, because his/her acts will be ignored and rendered irrelevant.

This study presents some limitations. First, this analysis excluded manuscripts and published articles available online, since DISCERN and JAMA were not developed to evaluate scientific information. Although laypersons can be interested in reading technical knowledge, the difficulties in understanding and interpreting those contents probably favour the consumption of accessible websites as analysed here. Second, for the same reason, images, videos and audios were not included in this analysis. Third, the statistics of access of each website were not verified to measure the real impact of specific information on internet users; however, data were collected only from links that were primarily retrieved by search engine tools, mimicking the usual behaviour of health seekers.^19^

## CONCLUSION

In conclusion, the quality of toothache-related information found in Brazilian websites was classified as simple, accessible and of low quality. With this in mind, professionals should be prepared to prescribe the consumption of good-quality health information for their patients, contributing to selection, interpretation and decision-making processes. In addition, public and private sectors should stimulate the production of digital health materials, since ICTs constitute a singular opportunity to disseminate information for the improvement of oral health status of distinct populations. As Foucault recognised, the first task of health professionals is political, since the struggle against disease has become a political and economic problem that demands a population-based approach, just as a war against bad government.^24,26^

Regarding specifically toothache, people should be adequately informed about its main causes and consequences, decreasing the chance of further painful episodes, the risk of dental treatment delays, and the costs of complicated clinical cases towards the advancement of a more effective healthcare system.
